# Stability of diluted chlorhexidine for skin testing in drug allergy evaluations

**DOI:** 10.1016/j.jacig.2024.100372

**Published:** 2024-11-26

**Authors:** Divya Shah, Gabriel Cojuc-Konigsberg, Stacy D. Brown, Sergio E. Chiarella, Gerald W. Volcheck, Hirohito Kita, Lene H. Garvey, Alexei Gonzalez-Estrada

**Affiliations:** a Department of Internal Medicine, University of Arizona College of Medicine, Phoenix, Ariz; b Medical School, Faculty of Health Sciences, Universidad Anahuac, Mexico City, Mexico; c Department of Pharmaceutical Sciences, East Tennessee State University, Johnson City, Tenn; d Division of Allergic Diseases, Mayo Clinic, Rochester, Minn; e Danish Anaesthesia Allergy Centre, Allergy Clinic, Department of Dermatology and Allergy, Copenhagen University Hospital-Herlev and Gentofte, Copenhagen, Denmark; f Department of Clinical Medicine, University of Copenhagen, Copenhagen, Denmark; g Division of Allergy, Asthma and Clinical Immunology, Mayo Clinic, Scottsdale, Ariz

**Keywords:** Chlorhexidine/adverse effects, drug hypersensitivity/diagnosis, humans, skin tests, drug stability

## Abstract

**Background:**

Chlorhexidine gluconate (CHX), a common cause of perioperative anaphylaxis, is frequently used for skin testing in allergy evaluations. Although CHX’s maximal nonirritating concentrations are known, the stability of its dilutions for skin testing remains unexplored, particularly when sterile water for injection (SWFI) or normal saline (NS) are used as diluents.

**Objective:**

Our aim was to evaluate the stability and precipitation of CHX when diluted with SWFI or NS for drug allergy skin testing.

**Methods:**

CHX dilutions (5-0.002 mg/mL) were prepared using SWFI and NS. HPLC and UV-visible spectrophotometry were used to assess stability and precipitation over 48 hours. Turbidity was measured at various time points to monitor precipitation.

**Results:**

HPLC analysis showed no significant differences in peak heights between CHX-SWFI and CHX-NS dilutions. However, visible precipitation and increased turbidity (>100 NTU) were observed in CHX-NS at higher concentrations (5 mg/mL) after 60 minutes. No precipitation occurred in CHX-SWFI at any concentration for 48 hours.

**Conclusion:**

For CHX skin testing, SWFI is the preferred diluent at concentrations higher than 0.02 mg/mL to avoid precipitation. Using NS for the final dilution from 0.02 to 0.002 mg/mL is feasible and reduces injection pain. Except for CHX-NS at 5 mg/mL, reagents can be prepared up to 24 hours before testing.

## Introduction

Chlorhexidine gluconate (CHX), a highly effective antiseptic and disinfectant, is a common cause of perioperative anaphylaxis and should always be included during perioperative anaphylaxis evaluations.^1^^,^^2^ Although studies have reported on the maximal nonirritating concentrations of dye-free CHX for skin testing(5 mg/mL for skin prick testing and 0.002 mg/mL for intradermal testing), the stability of diluted CHX for drug allergy skin testing remains unexplored.^1^^,^^3^^,^^4^ Because of early observations that precipitation occurred when normal saline was used at higher concentrations, in the Danish Anesthesia Allergy Centre, CHX is diluted with sterile water for injection at concentrations higher than 0.02 mg/mL. For CHX concentrations of 0.02 mg/mL, normal saline (NS) is used for the last step of dilution to the recommended dose of 0.002 mg/mL for intradermal testing, as injection is less painful than if sterile water for injection (SWFI) is used. This conclusion is based on anecdotal evidence, with several patients reporting that injections were more painful with SWFI before we switched to normal saline, as observed in unpublished observation (L.H. Garvey). These observations have never been confirmed experimentally; therefore, the objective of this study was to determine the stability of CHX diluted with either SWFI or NS used for skin testing.

## Results and discussion

We adapted a prior HPLC analytic detection method for diluted chlorhexidine. The analysis was performed using HPLC (Shimadzu, Kyoto, Japan) and an XDB C-18 column maintained at 40°C.^5^^,^^6^^,^^7^ The mobile phases included (A) 70% sodium phosphate buffer with triethanolamine and (B) 30% acetonitrile at a flow rate of 0.500 mL per minute for all chlorhexidine reagents.^5^ The HPLC variables sample injection (2 μL or 50 μL) and wavelength (239 nm) were optimized for each analyte.

**Supplementary Figure E1:**
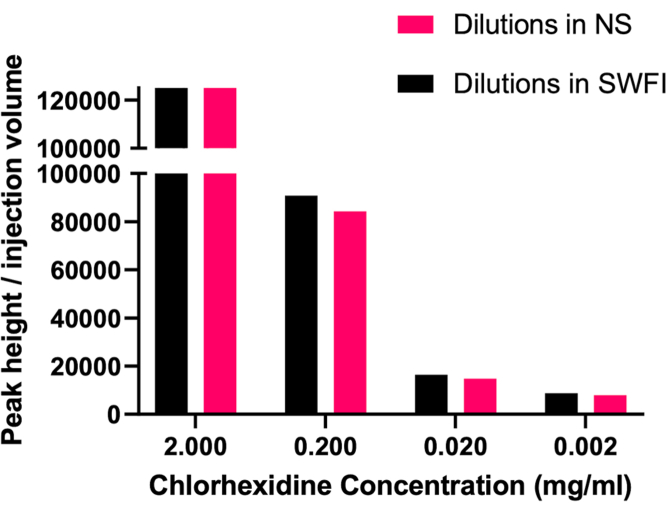


Ten-fold dilutions (2, 0.2, 0.02, and 0.002 mg/mL) of chlorhexidine (National Drug Code [NDC] identifier 0234-0575041) were prepared with 0.9% NS or SWFI into clear scintillation 20-mL vials and refrigerated (at 4°C). Initial peak height data were collected (see Fig E1 in the Online Reposiotry at www.jaci-global.org), but CHX-NS precipitation disallowed additional HPLC measurements on account of clogging in the instrument tubing. There was visible evidence of CHX-NS precipitation in the tubes at a concentration of 20 mg/mL left overnight (see Fig E2 in the Online Repository at www.jaci-global.org).

For additional investigation of precipitation, we used a handheld UV-visible spectrophotometer (Photopette, Tip Biosystems, Ayer Rajah Crescent, Singapore) to measure turbidity in a volume of 45 μL at a wavelength of 570 nm for 2 CHX dilutions: 5 mg/mL and 0.002 mg/mL. We prepared dye-free CHX (NDC identifier 0363-1061-08) dilutions (5 and 0.002 mg/mL) at a final volume of 1 mL with NS (NDC identifier 0409-4888-10), SWFI (NDC identifier 0409-4887-10), and SWFI+NS into 1.5-mL Eppendorf tubes (Fisher Scientific, Hampton, NH) at room temperature. Because SWFI can potentially irritate the skin of patients when injected intradermally, we added an analyte in a concentration of 0.002 mg/mL, which was diluted with SWFI for the first steps but with NS for the final dilution from 0.02 to 0.002 mg/mL (SWFI + NS). The 0.002-mg/mL analytes underwent 0.22-μm filtering (Merck Millipore, Cork, Ireland), as used in clinical practice for intradermal skin testing,^8^ A baseline measurement for each reagent was obtained immediately after preparation. After dilution at 5 mg/mL or 0.002 mg/mL, the samples were assessed for turbidity (nephelometric turbidity units [NTU]) in sextuplicate every 10 minutes for 60 minutes, then every hour for 24 hours, and once at 48 hours. Each sample was vortexed for 5 seconds before measurement. Precipitation was determined by visual observation; a turbidity measurement higher than 100 NTU was defined as precipitation-free.^9^ For data analysis, we compiled the measurements in GraphPad Prism 9.0 (GraphPad Software, Inc, San Diego, Calif) and calculated mean values with SDs.

**Fig 1:**
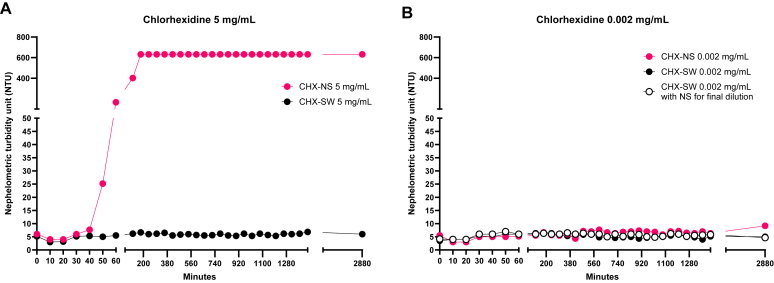
Turbidity of CHX at 5 mg/mL (**A**) and 0.002 mg/mL (**B**) during a 48-hour period with either NS (*pink circle*) or SWFI (*black circle*). **B,** A group of analytes that were diluted with SWFI to a concentration of 0.02 mg/mL were also diluted with NS for a final concentration of 0.002 mg/mL (*white circle*). The mean solution turbidity from measurements in sextuplicate is plotted in NTU for each time point.

The HPLC analysis showed no significant differences in peak heights between CHX dilutions in SWFI and NS (see Table E1 in the Online Repository at www.jaci-global.org). However, visible evidence of CHX-NS precipitation was observed at concentrations of 20 and 5 mg/mL despite the use of dye-free CHX at around 60 minutes. Our UV-visible results revealed that CHX-NS at 5 mg/mL presented an increased turbidity of more than 100 NTU around minute 60 but not at a concentration of 0.002 mg/mL (Fig 1). In contrast, at both concentrations (5 and 0.002 mg/mL), CHX-SWFI did not become turbid for at least 48 hours (Fig 1). The mean values (in NTU) and SDs for each reagent during the study period are presented in Table E1.

**Fig 2:**
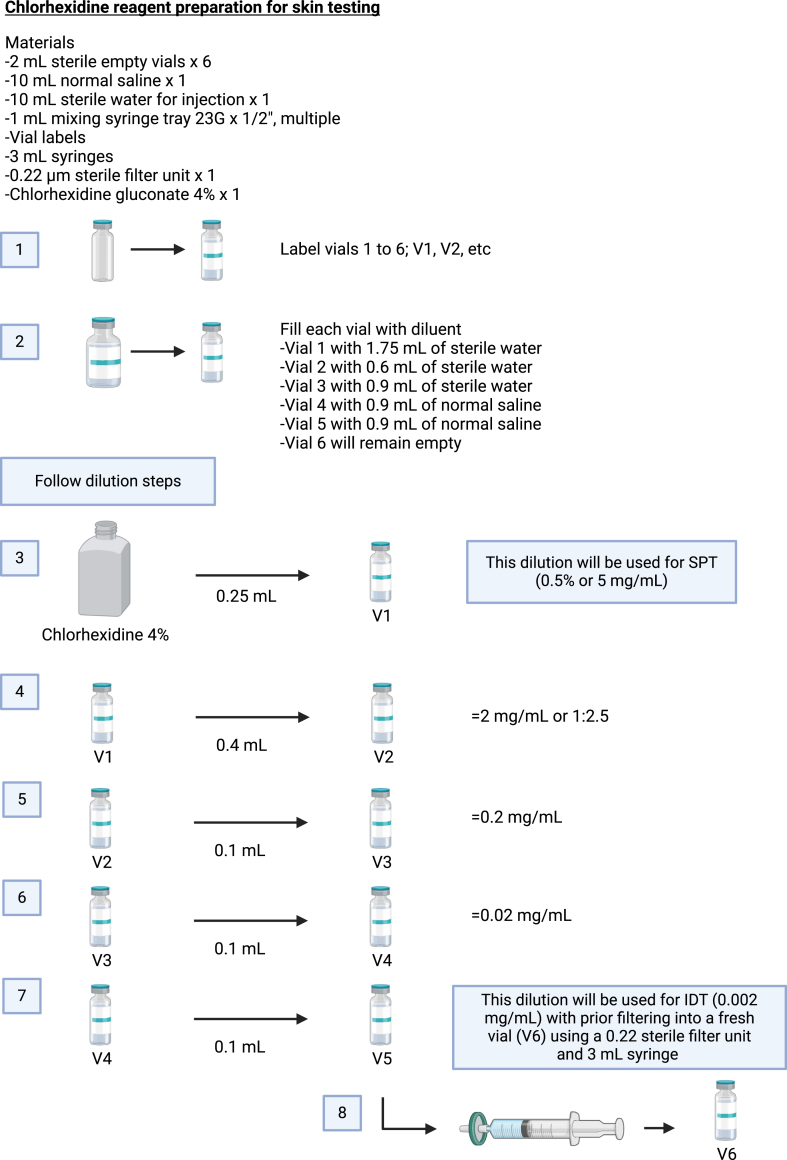
Proposed chlorhexidine reagent preparation for clinicians, including dilution for skin prick testing and for intradermal testing.

The observed findings support using sterile water for injection as the diluent of choice for chlorhexidine skin testing at concentrations higher than 0.02 mg/mL. We showed that at higher chlorhexidine (5 mg/mL) concentrations, a reaction with 0.9% normal saline is more likely to manifest a precipitate. Our analysis also suggests that using NS to dilute CHX at the last step of dilution from a concentration of 0.02 mg/mL to the concentration of 0.002 mg/mL recommended for intradermal skin testing is feasible and will reduce pain on injection. Our data also suggest that these reagents, with the exception of CHX-NS 5 mg/mL, may potentially be prepared 24 hours before CHX skin testing. An example protocol that is used at our institution to prepare these reagents for clinical practice is shown in Fig 2. This protocol should be adapted based on the final volumes for dilutions used by institutions and the initial stock concentrations of CHX, which vary geographically.^10^ One of this study’s limitations is that the results may not be generalizable to other chlorhexidine preparations.

In summary, our study suggests that whereas the CHX concentration of 0.002 mg/mL for intradermal testing can be prepared using either SWFI or NS without precipitation, the 5 mg/mL concentration for skin prick testing should be made and stored as SWFI, as precipitation was observed with NS at this concentration.

**Clinical implications**

To minimize precipitation risk during chlorhexidine dilution for skin testing, sterile water is advisable for concentrations higher than 0.02 mg/mL, whereas normal saline is suitable for diluting chlorhexidine concentrations of 0.02 mg/mL and lower.

## Disclosure statement

Funding was provided by the Arizona Department of Health Services ABRC Award (RFGA2023-008-27). The funds were destined to cover reagents. The funding organization had no role in the design and conduct of the study; collection, management, analysis, and interpretation of the data; preparation, review, or approval of the article; and decision to submit the article for publication.

Disclosure of potential conflict of interest: The authors declare that they have no relevant conflicts of interest.

## Supplementary data

Supplementary Figure E2

Supplementary Table E1
