# Supplementary figures and images for: Stability of diluted chlorhexidine for skin testing in drug allergy evaluations
Source: J Allergy Clin Immunol Glob. 2024 Nov 26;4(1):100372. doi: 10.1016/j.jacig.2024.100372 (PMC11719288; doi:10.1016/j.jacig.2024.100372)

**A)**

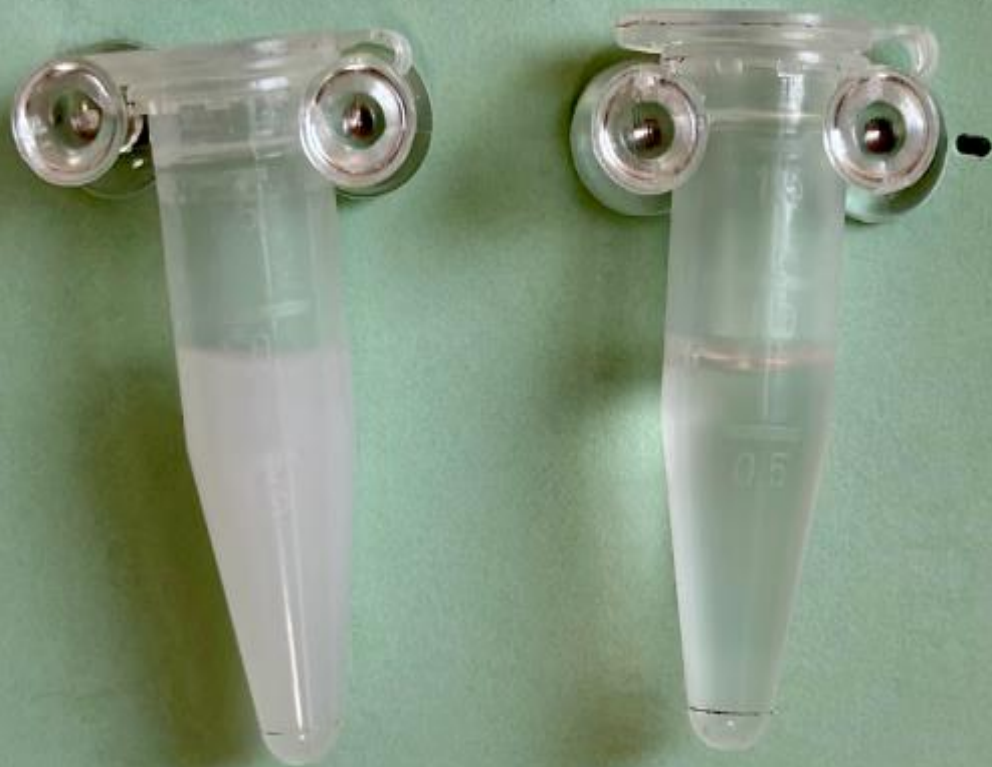

**CHX-NS**

**CHX-SWFI**

**B)**

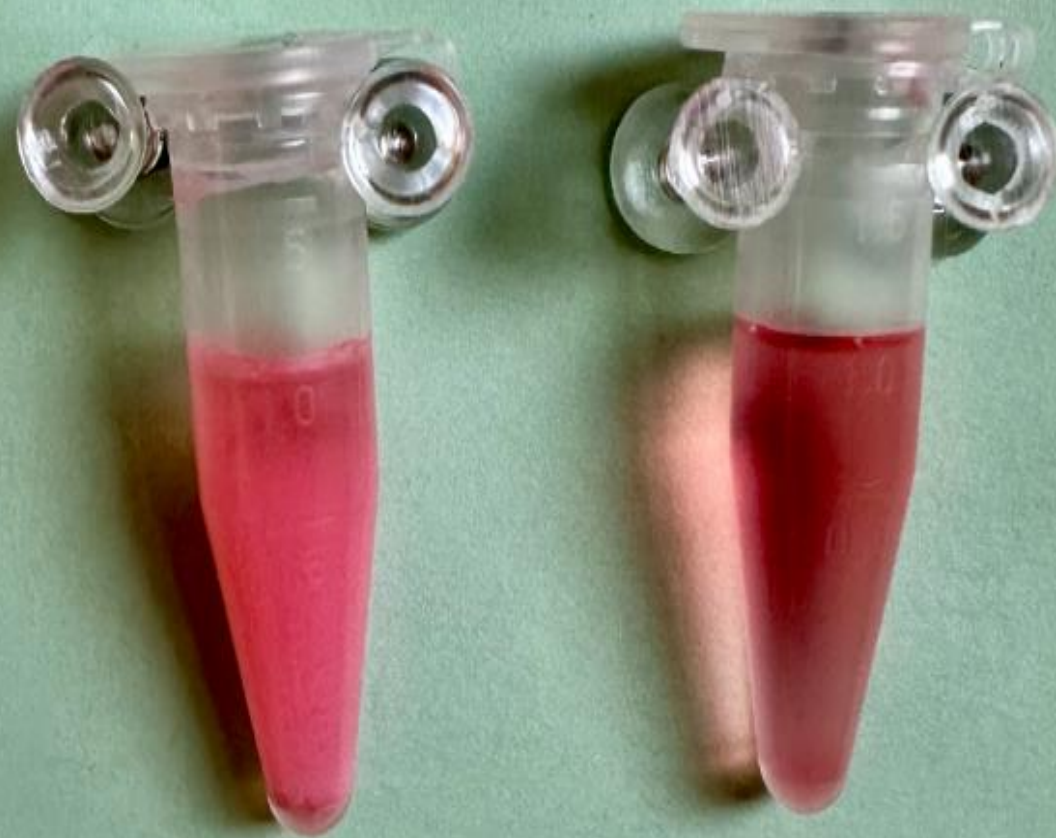

**CHX-NS**

**CHX-SWFI**

Supplement: Supplementary Figure E2 [file mmc1.pdf]
