# Supplementary material for: Stability of diluted chlorhexidine for skin testing in drug allergy evaluations
Source: J Allergy Clin Immunol Glob. 2024 Nov 26;4(1):100372. doi: 10.1016/j.jacig.2024.100372 (PMC11719288; doi:10.1016/j.jacig.2024.100372)
Supplement: Supplementary Table E1 [file mmc2.docx]

| **Table E1.** Mean (±SD) nephelometric turbidity units of chlorhexidine dilutions | | | | | | |
| --- | --- | --- | --- | --- | --- | --- |
|  | **CHX-NS** | |  | **CHX-SWFI** | | |
| **Time in minutes (hours)** | **5 mg/mL** | **0.002 mg/mL** |  | **5 mg/mL** | **0.002 mg/mL** | **0.002 mg/mL with last NS dilution** |
| 0 (0) | 6±0 | 5.5±0.5 |  | 5.2±0.4 | 3.5±0.5 | 4.2±0.4 |
| 10 (0.16) | 4±0 | 3±0 |  | 3±0 | 4±0 | 4±0 |
| 20 (0.33) | 4±0 | 3±0 |  | 3.2±0.4 | 4±0 | 4±0 |
| 30 (0.5) | 6±0 | 5±0 |  | 5.2±0.4 | 5.3±0.5 | 6±0 |
| 40 (0.66) | 7.7±1.1 | 5±0 |  | 5.3±0.5 | 5±0 | 6±0 |
| 50 (0.83) | 25.2±2.5 | 5±0 |  | 5±0 | 6±0 | 7±0 |
| 60 (1) | 166.8±39.7 | 5.2±0.4 |  | 5.5±0.8 | 6±0 | 6±0 |
| 120 (2) | 402±18.4 | 5.5±1.1 |  | 6.2±0.7 | 6.3±0.5 | 6±0 |
| 180 (3) | 632±0 | 6.7±1.5 |  | 6.7±0.5 | 6.7±0.5 | 6.3±0.5 |
| 240 (4) | 632±0 | 5.7±1.5 |  | 6±0 | 6.3±0.5 | 6±0 |
| 300 (5) | 632±0 | 5.5±1.6 |  | 6.2±0.4 | 5.5±1.1 | 6±0 |
| 360 (6) | 632±0 | 6±0.6 |  | 6.5±0.5 | 5.3±0.5 | 6.5±0.5 |
| 420 (7) | 632±0 | 4.3±0.7 |  | 5.5±1.1 | 5.2±0.7 | 6±0 |
| 480 (8) | 632±0 | 7.2±0.7 |  | 5.8±0.7 | 5.8±0.4 | 6.3±0.5 |
| 540 (9) | 632±0 | 7±0.6 |  | 6±0 | 5.8±0.4 | 6±0 |
| 600 (10) | 632±0 | 7.7±0.5 |  | 5.7±0.5 | 4.8±0.7 | 6±0.6 |
| 660 (11) | 632±0 | 6.7±1.1 |  | 5.5±0.5 | 5±0 | 5±0 |
| 720 (12) | 632±0 | 6.3±1.1 |  | 5.6±0.5 | 4.6±0.5 | 6±0.8 |
| 780 (13) | 632±0 | 6.8±0.4 |  | 6.2±0.7 | 4.8±0.7 | 5.2±0.4 |
| 840 (14) | 632±0 | 7±1.3 |  | 5.5±0.8 | 5±0.8 | 6±0.6 |
| 900 (15) | 632±0 | 7.3±1.2 |  | 5.3±0.7 | 4.3±0.7 | 5.8±0.7 |
| 960 (16) | 632±0 | 7±0.8 |  | 6.2±0.7 | 4.7±0.5 | 5±0 |
| 1020 (17) | 632±0 | 6.8±1.5 |  | 5.3±0.5 | 5.2±0.7 | 4.8±0.4 |
| 1080 (18) | 632±0 | 5.8±1.1 |  | 6.2±0.4 | 5±1.2 | 5.2±0.7 |
| 1140 (19) | 632±0 | 7±0 |  | 5.7±0.5 | 5.5±1.1 | 6.2±0.4 |
| 1200 (20) | 632±0 | 7.2±0.9 |  | 5.3±0.5 | 5.8±0.4 | 6±0 |
| 1260 (21) | 632±0 | 6.5±0.5 |  | 6.2±0.7 | 5±0 | 5.2±0.4 |
| 1320 (22) | 632±0 | 6.3±0.7 |  | 6±0 | 4.8±0.7 | 5.5±1.3 |
| 1380 (23) | 632±0 | 7±1 |  | 6.2±0.4 | 4±0 | 5.7±0.5 |
| 1440 (24) | 632±0 | 6.3±0.5 |  | 6.8±0.7 | 5.2±0.9 | 5.8±0.9 |
| 2880 (48) | 632±0 | 9.2±0.4 |  | 6±0 | 5±0.6 | 4.7±1.1 |
| Abbreviations: SD, standard deviation; CHX, chlorhexidine; SWFI, sterile water for injection; NS, 0.9% normal saline. | | | | | | |
